# The Relation of Parental Emotion Regulation to Child Autism Spectrum Disorder Core Symptoms: The Moderating Role of Child Cardiac Vagal Activity

**DOI:** 10.3389/fpsyg.2018.02480

**Published:** 2018-12-14

**Authors:** Xiaoyi Hu, Zhuo Rachel Han, Hui Wang, Yannan Hu, Qiandong Wang, Shuyuan Feng, Li Yi

**Affiliations:** ^1^Department of Special Education, Education Research Center for Children with ASD, Faculty of Education, Beijing Normal University, Beijing, China; ^2^Beijing Key Laboratory of Applied Experimental Psychology, National Demonstration Center for Experimental Psychology, Faculty of Psychology, Beijing Normal University, Beijing, China; ^3^School of Psychological and Cognitive Sciences, Peking University, Beijing, China

**Keywords:** autism spectrum disorders, emotion regulation, core symptoms, cardiac vagal activity, heart rate variability, parenting

## Abstract

This study investigated the role of parental emotion regulation (ER) on children’s core symptoms in families of children with autism spectrum disorders (ASD) in middle childhood; the study also explored whether children’s physiological ER functioning served as a risk or protective factor with respect to parental relationships. Thirty-one Chinese children with ASD (age 6–11) and their primary caregivers participated in this study. Parental ER and child ASD symptoms were collected via questionnaires from parents. Child cardiac vagal activity (derived from heart rate variability) was measured at rest and during a parent-child interaction task. Using moderation analyses, the results showed that parental ER was not directly associated with children’s core ASD symptoms; rather, it interacted significantly with children’s resting cardiac vagal activity, but not task-related changes of cardiac vagal activity, to exert an impact on children’s core ASD symptoms. Specifically, our findings suggested that parents’ difficulties with their own ER significantly impacted their children’s core ASD symptoms only for the children who showed blunted resting cardiac vagal activity. Implications for the future measurement of ER in the family context and future directions for intervention are discussed.

## Introduction

Autism spectrum disorder (ASD) is a lifelong pervasive neurodevelopmental disorder characterized by deficits in social communication and social interaction as well as restricted, repetitive patterns of behavior, interest, or activities (American Psychiatric Association, 2013). Many individuals with ASD experience tremendous challenges in modulating their emotional responses and coping with depression, anxiety, aggression, tantrums, or self-injury (Weiss, 2014; Chandler et al., 2015; Mazefsky, 2015). These problems can significantly impact the development and quality of life for children with ASD, as well as the psychological wellbeing of their parents (Boonen et al., 2014; Ting and Weiss, 2017). As such, researchers and practitioners have begun to pay substantial attention to factors that can influence the changes in emotion related factors of children with ASD such as social communication skills, restricted interests, and repetitive behaviors (e.g., Zachor et al., 2007).

Among the potential influencing factors are parents’ own emotional characteristics, setting the emotional tone of the dyadic relationships between parents and children (Morris et al., 2017), which may be particularly important for the overall functioning of children with ASD, vis-a-vis their core symptoms. Indeed, parents’ own emotional characteristics, especially parental emotion regulation (ER), have long been considered by developmental psychologists as integral to a child’s development of emotional and social competence and functioning (Dix, 1991). ER refers to the internal and external processes that including initiating, evaluating, maintaining and modifying emotional reactions to meet situational demands (Thompson, 1994; Gross, 2014). Parental ER is considered a direct method through which parents might influence children’s socio-emotional functioning (Morris et al., 2007). In typically developing children, parental ER has been associated with a series of children’s developmental outcomes, especially psychopathological symptoms (e.g., Han and Shaffer, 2013) and socio-emotional functioning (e.g., Silk et al., 2006). However, we know little regarding how the ER of parents of children with ASD might influence the core symptoms of their offspring. Due to their children’s autistic symptom, parents of children with ASD could experience tremendous stress in parenting, giving prominence to the role of emotional regulation.

Despite no study having directly assessed the relationship between parental ER and core symptoms of children with ASD, some studies have demonstrated that interventions targeting parental emotional responsiveness and emotional bonding relationship (e.g., rapport building, emotional reciprocity, affect sharing) can improve children’s social-emotional functioning and communication skills, which are the fundamental deficits in ASD (e.g., Mahoney and Perales, 2005; Kasari et al., 2006; Stahmer et al., 2011). In a recent randomized controlled trial (RCT) study implementing parent-implemented reciprocity treatment to address joint and emotional interactive relationship building through playing activities for a total of 22 children with ASD between 2 and 6 years old, the outcomes showed improvement in core ASD symptoms (Gengoux et al., 2018). Together, these primary findings implied that parental emotionality might be particularly important for the changes of core symptoms in children with ASD. Additionally, previous research on parental ER and the psychopathological symptoms in typically developing children also note that not all children whose parents have problems with ER eventually develop maladaptive symptoms (e.g., Han and Shaffer, 2013). This is consistent with the risk and resilience model and the diathesis-stress model which suggest that a risk (or a stress) appears to have differential predictive effects on child outcomes depending on the level of a protective or vulnerability factor (or a diathesis) (see Masten, 2001 for a review). Based upon this risk and resilience framework, it is likely that some children with ASD may be more resilient to the deleterious effect of dysregulated parental emotions than others. Similarly, not all children with parents who are better at regulating their own emotions are free of psychopathological symptoms. These possible discrepancies between parental emotion dysregulation and the overall ASD children’s outcomes warrant a search for protective or vulnerability factors.

Children’s own ER might be such a factor. In general, most research on ER in ASD has focused on the emotional experiences and ER weaknesses of high-functioning children or adolescents with ASD by comparing them to the typically developing individuals (Garon et al., 2009; Mazefsky et al., 2014; Samson et al., 2015). Children’s ER weaknesses might impact their social-emotional functioning which is critical to the core symptom severity of ASD (Mazefsky, 2015; Patel et al., 2017), regardless of how their parents regulate emotions. However, given that parents of children with ASD are more likely to have ER difficulties (Ting and Weiss, 2017), the combination of parental and children’s emotion dysregulation might amplify the core symptoms of ASD. Additionally, similar to the lack in exploring the relation between parental ER and child children’s ASD core symptoms, the examination of the possible moderating effect of child emotion dysregulation on this process has also been limited in the current literature.

More importantly, the majority of these studies on child ER in ASD research adopted self-report measures and behavioral observation to assess child ER. Researchers have long advocated the use of more physiological methods to evaluate children’s ER due to its multifaceted nature (e.g., Weiss et al., 2014). The functioning of the autonomic nervous system (ANS) is an important factor to consider in terms of fully understanding children’s emotional functioning (Bunford et al., 2017). The ANS has bidirectional signals passing through its two branches, the sympathetic nervous system (SNS) and parasympathetic nervous system (PNS), with the functioning of the PNS frequently being examined in psychophysiological studies on emotional and behavioral regulation (e.g., Calkins and Dedmon, 2000). The vagal nerve is the main nerve of the PNS (Laborde et al., 2017) and the cardiac vagal activity reflects the parasympathetic influence on the heart (Porges, 2007). Cardiac vagal activity has often been estimated by heart rate variability (HRV) which refers the oscillations in the interval between consecutive heartbeats (Camm et al., 1996). The polyvagal theory posits that resting cardiac vagal activity and the task-related changes of cardiac vagal activity in response to stress or emotionally challenging events reflect ER and bio-behavioral flexibility (Porges, 2007). However, they are differentially related to physiological and emotional processes (Beauchaine, 2001; Rottenberg et al., 2005), although they are sometimes found to be related to one another (e.g., Patriquin et al., 2013b).

Research has shown that cardiac vagal activity may work as a physiological indicator for individuals’ capacity for self-regulation and adaptive functioning (Weber et al., 2010; Graziano and Derefinko, 2013; Laborde et al., 2017). Specifically, resting cardiac vagal activity is thought to reflect autonomic flexibility and a relatively stable, trait-like capacity for ER, whereas the degree of cardiac vagal activity under stressful situations is theorized to be related to self-regulatory efforts and dynamic regulation of emotion (Beauchaine, 2001). During resting states, typically developing children’s high levels of cardiac vagal activity reflected their adaptive cardiovascular responses and was related to flexible ER (Fabes and Eisenberg, 1997), fewer internalizing symptoms (El-Sheikh et al., 2011; Borelli et al., 2015; Quiñones-Camacho and Davis, 2018), and decreased externalizing behaviors (e.g., El-Sheikh et al., 2011). Similarly, under challenging circumstances, children’s decreased cardiac vagal activity was viewed as one way organisms were mobilizing resources to deal with the stress and was associated with effortful ER (Gentzler et al., 2009; Cui et al., 2015), fewer anxiety and depression symptoms (Gentzler et al., 2012; El-Sheikh et al., 2013), and fewer externalizing symptoms (Lunkenheimer et al., 2017). Thus, the regulation of PNS and its optimal functioning should manifest as high levels of resting cardiac vagal activity and a reduction of cardiac vagal activity in response to stressful conditions, which working as indicators of physiological ER may subsequently be related to less psychopathological symptoms.

However, the studies examining the relationship between ASD children’s cardiac vagal activity and the severity of ASD symptoms have generated mixed findings; although a significant portion of the literature has linked low levels of cardiac vagal activity at rest and under pressure with increased ASD symptoms, other research has not. For instance, resting cardiac vagal activity has been found to be negatively associated with emotion recognition (Bal et al., 2010), restricted repetitive behaviors (Condy et al., 2017), and social interactions (Neuhaus et al., 2014; Patriquin et al., 2014), all of which are the core deficits and symptoms of severity of ASD. Other research, however, finds no relationship between resting cardiac vagal activity and social responsiveness of children with ASD (e.g., Patriquin et al., 2013a; Guy et al., 2014). With respect to cardiac vagal activity under pressure, some studies have reported that a small reduction of cardiac vagal activity correlate with increased social problems (Watson et al., 2010; Sheinkopf et al., 2013; Edmiston et al., 2016; Neuhaus et al., 2016), and increased restricted repetitive behaviors (Condy et al., 2017). Nonetheless, other studies have found null results (e.g., Kushki et al., 2014).

Despite the mixed results in existing literature, the preponderance of findings across cardiac vagal activity and the severity of ASD symptoms suggest that low resting cardiac vagal activity and a lack of suppressed cardiac vagal activity in response to challenges are likely indicative of different aspects of physiological dysregulation of emotion, which might amplify the negative impact of parental emotion dysregulation. Similarly, for those children with high resting cardiac vagal activity and adaptive suppression of cardiac vagal activity in response to the challenging environment, such physiological indicators of adaptive ER might buffer them from the potentially deleterious effect of parental emotion dysregulation. Thus, we examined the different roles of cardiac vagal activity at rest and during pressure in the association between parental ER and the core symptoms of children with ASD.

The current study focused on parental ER in families of children with ASD in middle childhood. We utilized both psychological and physiological methods to investigate the relationship among overall ER functioning in parents that sets the emotional tone of the parent–child relationship, children’s cardiac vagal activity at rest and task-related changes that indicates their physiological ER, and children’s core ASD symptoms. Specifically, it aimed to address the following questions: (1) Is parental behavioral ER associated with the core symptoms of children with ASD? (2) Does the relationship between parental behavioral ER and child core symptoms depend on differences in children’s trait-like physiological ER (i.e., resting cardiac vagal activity)? (3) Does children’s conditional physiological ER (i.e., task-related changes of cardiac vagal activity) moderate the association between parental behavioral ER and children’s core ASD symptoms? In the light of both theoretical considerations and relevant research findings, we hypothesized that children with ASD would exhibit more core symptoms if their parents had more difficulties with behavioral ER. We also hypothesized that high resting cardiac vagal activity and increased suppression of cardiac vagal activity in response to social stress would serve as protective factors on the relationship between parental ER difficulties and children’s core symptoms. Specifically, we hypothesized that under the condition of elevated parental emotion dysregulation, children with better trait-like and conditional physiological ER would be less likely to develop more core ASD symptoms than their counterparts who showed poor trait-like and conditional physiological ER. In contrast, under the condition of lower parental emotion dysregulation, the former group would be more likely to have decreased core ASD symptoms than the latter.

## Materials and Methods

### Participants

Thirty-one children with ASD between 6 and 11 years old (26 boys and 5 girls) and their primary caregivers (26 biological mothers and 5 biological fathers) were recruited in the current study via advertisements distributed in local community and online. The children have all been examined by experienced clinicians and met the diagnostic criteria for ASD according to the fourth edition of the *Diagnostic and Statistical Manual of Mental Disorders* (DSM-IV; American Psychiatric Association, 2000). Cognitive developmental abilities were evaluated by the Wechsler Abbreviated Scale of Intelligence for Children, Fourth Edition (WASI-IV; Wechsler, 2003). Two children were excluded because they were unable to complete the tasks. Thus, the final sample consisted of 29 children with ASD (*M age* = 8.00 years; *SD* = 1.51 years; 25 boys) and their parents. The mean of children’s full-scale IQ scores was 90.01 (*SD* = 22.07). Parents’ ages ranged from 33 to 47 years (*M* = 39.48, *SD* = 3.19). The primary parents were well educated; specifically, eight parents (25.8%) had completed graduate-level education, 20 (64.5%) had earned college degrees, and three (9.7%) reported earning a high school diploma. With respect to family income, 13.8% of the families lived with the household income of less than 120,000 RMB (approximately 18,000 USD), 41.4% of the families between 120,000 RMB (Chinese dollar) and 240,000 RMB, and 44.8% of the families greater than 240,000 RMB per year.

### Procedures

All study procedures were approved by the Institutional Review Board (IRB) of Faculty of Psychology, Beijing Normal University. Parent-child dyads were invited to visit the university laboratory for this study. Upon arrival at the lab, parents and children were informed of the purpose and procedures and signed informed consent forms and minor assent forms. Next, children and parents were fitted with physiological equipment. Specifically, three electrocardiogram (ECG) electrodes were attached to the participant’s abdomen under the left lower rib (+), chest below the right collarbone (-), and abdomen under the left lower rib (ground), based on the Einthoven’s triangle (Lead II; Biopac Systems Inc., 2016). Simultaneously, a respiratory belt was wrapped around the participant’s chest to record respiration (RSP) data. After attaching electrodes, a long adaptation period, approximately half an hour, occurred to support the ASD children to accommodate to the laboratory environment. Then, there occurred two 2-min resting sessions in which they were instructed to sit relaxed and breathe regularly without speaking or moving. Afterward, parent–child dyads participated in a dyadic collaboration task for 4 min, during which the dyads were asked to collaboratively draw a house and a tree using the classic drawing toy, Etch-a-Sketch. Each member of the dyad was instructed to use only one knob to control the horizontal or the vertical movement of the lines. This task is considered challenging for children with ASD, as it requires the constant and moment-to-moment collaboration between them and their parents. Additionally, as the relationship between cardiac vagal activity and emotional processes is supposed to be more robust in the context of social engagement (Butler et al., 2006), combined with the time limit and the requirement of social communication, this task was believed to be appropriate to understand the functioning of PNS under social stress for children with ASD. During the resting session and the dyadic collaboration task, the ECG and RSP signals of both parents and children were recorded. As the aim of the current study was to examine the moderating role of child physiological ER (i.e., child resting cardiac vagal activity and cardiac vagal activity suppression) on parental behavioral ER and child core symptoms, only children’s ECG and RSP data were used. Finally, we assessed all children with the abbreviated WISC-IV. Meanwhile, parents were asked to complete a packet of questionnaires in a separate room.

### Measures

#### Parental Emotion Dysregulation

The parents’ self-reports of difficulties in regulating their own emotions were collected via the Difficulties in Emotion Regulation Scale (DERS; Gratz and Roemer, 2004). The DERS is a 36-item measure that assesses current and clinically relevant difficulties with ER. The parents indicated how often the items apply to them using a 5-point Likert scale ranging from 1 (almost never) to 5 (almost always). The questionnaire consists of six subscales: non-acceptance of emotional responses (e.g., “When I’m upset, I become angry with myself for feeling that way”), difficulties engaging in goal-directed behavior (e.g., “When I’m upset, I have difficulty focusing on other things”), impulse control difficulties (e.g., “I experience my emotions as overwhelming and out of control”), lack of emotional awareness (e.g., “When I’m upset, I acknowledge my emotions”; reverse scored), limited access to ER strategies (e.g., “When I’m upset, I believe there is nothing I can do to make myself feel better”), and lack of emotional clarity (e.g., “I have no idea how I am feeling”). Scores of the subscales were summed to create an overall total score, and higher scores represented more difficulties in regulating one’s own emotions. Generally, the DERS shows good psychometric properties with high reliability and acceptable validity (Gratz and Roemer, 2004) and the Chinese version also demonstrates good psychometric properties (Li et al., 2018). The total score was used as the measure of parental emotion dysregulation for the present study (α = 0.82).

#### Autism-Related Characteristics

Parents completed the 50-item Autism-spectrum Quotient (AQ; Baron-Cohen et al., 2001) based on their children’s personalities and typical behaviors. The AQ uses a 4-point Likert-type scale (1 = “definitely disagree,” 2 = “slightly disagree,” 3 = “slightly agree,” 4 = “definitely agree”) to screen autism-related traits. It consists of five subscales (10 items each) to assess social skills (e.g., “He/she finds it hard to make new friends”), attention switching (e.g., “He/she prefers to do things the same way over and over again”), attention to detail (e.g., “He/she often notices small sounds when others do not”), communication (e.g., “He/she is often the last to understand the point of a joke”), and imagination (e.g., “He/she finds it difficult to imagine what it would be like to be someone else”). The overall score ranging from 50 to 200 was used to reflect the severity of autistic symptoms. Children who exhibited more autism-related characteristics generally got higher scores. The original English version of AQ shows satisfactory reliability and validity (Baron-Cohen et al., 2001), and the Chinese version also has good psychometric qualities (Auyeung et al., 2008; Zhang et al., 2016). In the current study, the internal consistency of the AQ total score was acceptable (α = 0.829).

#### Child HRV

The ECG and RSP signal were monitored and recorded using a 16-channel physiological recorder (BIOPAC MP150, Biopac Systems Inc., 2016). The amplifier gain was set to 2000, with the high-pass filter set to 0.5 Hz, the low-pass filter set to 35 Hz, and the sampling frequency set to 1000 Hz. Prior to analyses, the ECG data were inspected and inter-beat intervals were visually checked and edited for artifacts using the MindWare HRV 3.1.1 program (Mindware Technologies, Ltd., Gahanna, OH, United States). In this study, high-frequency heart rate variability (HF-HRV) was used as previous theory and research indicated it reflected parasympathetic-mediated control over the heart and could be worked as an indicator of cardiac vagal activity (Porges, 2007; Thayer et al., 2009; Gerritsen and Band, 2018). The HF-HRV was calculated for every 30-s epoch by spectral-analyzing the R-wave time series of the ECG within the frequency band at childhood (i.e., 0.24–1.04 Hz, Camm et al., 1996; Berntson et al., 2007). The children’s resting HF-HRV was established by averaging the four 30-s epochs during the resting session. The children’s HF-HRV reactivity was calculated by subtracting task-related HF-HRV from resting HF-HRV. Thus, positive scores on HRV reactivity represented a decrease of cardiac vagal activity in response to dyadic collaboration task compared to the resting state (e.g., Calkins et al., 2008). The missing rate of HRV epoch values was 15.71% mainly due to autistic children’s sensitive skin, equipment failure, poor signal quality or research assistant error.

### Data Analysis

Prior to analysis, a missing value analysis was conducted in SPSS 21.0. The amount of missing data was relatively low across study variables (ranged between 0% and 17.2%), with most due to participants’ failure to complete the measures and equipment failure. Little’s (1988) missing completely at random (MCAR) test revealed that data was MCAR, χ^2^ (12, *N* = 29) = 14.506, *p* = 0.27. We therefore input missing data using the expectation-maximization algorithm as has been recommended for analyses that meet the MCAR assumptions (Howell, 2007).

Next, the preliminary analyses evaluating the descriptive statistics, correlations among the variables of interest, and possible group differences in study variables based on demographic characteristics were performed. Finally, the moderation models, with child baseline HF-HRV and HF-HRV reactivity as moderators, on the association between parental emotion dysregulation and child autism-related characteristics were explored using the PROCESS SPSS macro (Hayes, 2012) with a bootstrap resample of 5000. Specifically, template model 1 of this PROCESS macro was used. Because of the potential influence of respiration on HF-HRV magnitude and confound HF-HRV as an accurate estimate of cardiac vagal activity (Berntson et al., 2007; Laborde et al., 2017), children’s respiration rates during resting period and the collaboration task were included in the moderation models as covariates. In addition, any demographic (i.e., child age and sex, parent age) or intellectual (i.e., child IQ) factor that was associated with the outcome variable was controlled in the analysis. Moderation was valid when the interaction term between the predictor and the moderator variable was significant and the confidence interval did not include 0. We used 95% confidence intervals, and the conditional relationship between the independent and dependent variables was examined at low (-1 SD below the mean) and high (+1 SD above the mean) levels of the moderator variables (i.e., child baseline cardiac vagal activity and cardiac vagal activity suppression).

## Results

### Preliminary Analyses

The results of preliminary descriptive statistics of all study variables are presented in Table 1. Child autism-related characteristics were negatively correlated with their own resting HF-HRV (*r* = -0.57, *p* < 0.01), suggesting that children who exhibited lower resting HF-HRV presented more autistic traits. Parental emotion dysregulation was positively related to children’s resting HF-HRV (*r* = 0.43, *p* < 0.05), but not related to HF-HRV reactivity during the task. However, parental emotion dysregulation was not found to be significantly correlated with children’s autism-related characteristics.

**Table 1:** Means, standard deviations, and correlations among studied variables.

Variable	*M*	*SD*	1	2	3
(1) P-DERS	80.79	14.59			
(2) C-Resting HRV	5.91	1.02	0.43*		
(3) C-HRV Reactivity	0.29	0.63	0.06	0.14	
(4) AQ	89.79	18.91	-0.01	-0.58**	-0.05


*P-DERS, Parental emotion dysregulation; C-Resting HRV, Child resting heart rate variability; C-HRV Reactivity, Task-related changes of child heart rate variability. ^∗^*p* < 0.05, ^∗∗^*p* < 0.01.*

Next, we examined the possible group differences in all study variables. Results demonstrated that none of the demographic characteristics (i.e., child age, child gender, and parent age) and children’s cognitive abilities (i.e., full-scale IQ) were significantly correlated with any study variables. However, in line with other studies (e.g., Staton et al., 2009; Saghir et al., 2017), we controlled the influences of participant demographics and children’s full-scale IQ in further analyses, as ASD children’s HRV and the severity of their ASD symptoms might be varied by these factors (e.g., Condy et al., 2017).

### Moderation Model

The moderation analysis examined whether child resting HF-HRV and HF-HRV reactivity moderated the link between parental emotion dysregulation and child autistic traits. Applying the PROCESS SPSS macro for moderation analyses, the conditional effect of parental emotion dysregulation was estimated at two values of the dichotomized levels of child resting HF-HRV and HF-HRV reactivity.

**FIGURE 1:**
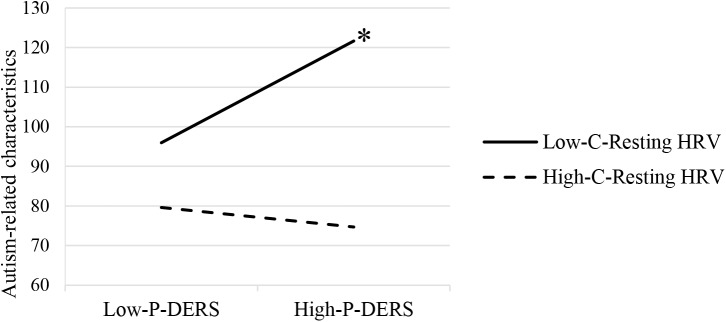
Moderating effects of child resting HRV on the relationship between parental emotion dysregulation and ASD child autism-related characteristics. P-DERS, Parental emotion dysregulation; C-Resting HRV, Child resting heart rate variability. ^∗^*p* < 0.05.

The moderation analysis with child resting HRV as a moderator showed that child resting HF-HRV significantly moderated the relationship between parent’s self-reported difficulties in ER and parental report of child autism-related characteristics (*R^2^* change = 0.10, *p* = 0.036). Moderation effects have also been found after controlling the influences of participant demographics and child IQ and respiration rates during resting period (*R^2^* change = 0.11, *p* = 0.037; see Table 2). As shown in Figure 1, the conditional effect estimates further demonstrated that parental emotion dysregulation was positively associated with children’s autism-related characteristics among ASD children with lower resting HRV, *b* = 0.84, *p* = 0.01. However, among ASD children with relatively higher resting HF-HRV, their parents’ emotion dysregulation was not significantly related to their autism-related characteristics. It appeared that parental emotion dysregulation was positively related to the severity of symptoms in ASD children but only for children who exhibited lower resting HRV.

**Table 2:** Results of moderation analysis on child autism-related characteristics.

Outcome variable	*b* (SE)	*t*	*R^2^*	*F*	95% CI
**AQ**			0.56	3.18^∗^	
P-DERS	3.56 (1.44)	2.47*			0.55, 6.56
C-Resting HRV	27.29 (18.93)	1.44			-12.19, 66.78
C-Resting RR	0.84 (0.98)	0.86			-1.21, 2.89
Child age	-1.05 (2.60)	-0.40			-6.48, 4.37
Child sex	-4.84 (9.87)	-0.49			-25.43, 15.76
Parent age	-0.99 (0.99)	-1.00			-3.07, 1.08
Full-scale IQ	0.01 (0.98)	0.86			-1.21, 2.89
DERS × C-Resting HRV	-0.54 (.24)	-2.24*			-1.04, -0.04
- 1 SD (C-Resting HRV)	0.88 (0.32)	2.77*			0.22, 1.54
+1 SD (C-Resting HRV)	-0.17 (.33)	-0.52			-0.85, 0.51
**AQ**			0.20	0.53	
P-DERS	-0.22 (0.39)	-0.57			-1.05, 0.60
C-HRV reactivity	-68.45 (47.94)	-1.43			-168.81, 31.91
C-Resting RR	-1.07 (1.27)	-0.85			-3.73, 1.59
C-RR during task	0.56 (1.40)	0.40			-2.36, 3.49
Child age	-0.28 (3.87)	-0.07			-08.39, 7.82
Child sex	3.02 (13.63)	0.22			-25.51, 31.54
Parent age	-2.19 (1.37)	-1.60			-5.06, 0.67
Full-scale IQ	-0.09 (.25)	-0.37			-0.63, 0.44
P-DERS × C-HRV reactivity	0.75 (0.53)	1.42			-0.36, 1.87
-1 SD (C- HRV reactivity)	-0.46 (0.52)	-0.90			-1.55, 0.62
+1 SD (C- HRV reactivity)	0.60 (0.45)	1.32			-0.35, 1.54


*P-DERS, Parental emotion dysregulation; C-Resting HRV, Child resting heart rate variability; C-HRV Reactivity, Task-related changes of child heart rate variability; C-Resting RR, Child resting respiration rate; C-RR during task, Child respiration rate during the interaction task. ^∗^*p* < 0.05, ^∗∗^*p* < 0.01, ^∗∗∗^*p* < 0.001.*

Additionally, the moderation analysis with child HF-HRV reactivity as a moderator demonstrated that the change of child HRV from resting to a stressful task was not a significant moderator for the relationship between parental emotion dysregulation and child autism-related characteristics (see Table 2).

## Discussion

Based upon the risk and resilience framework (Masten, 2001), the overarching goal of the current study was to examine, in families of children with ASD, the role of children’s physiological ER functioning (the resting cardiac vagal activity that reflects the trait-like physiological ER and the cardiac vagal activity to stressful interaction that reflects the conditional physiological ER) on the relationship between parental emotion dysregulation and children’s core ASD symptoms.

We found that although parental emotion dysregulation was not directly associated with children’s core ASD symptoms, it interacted significantly with children’s resting cardiac vagal activity (but not task-related changes of cardiac vagal activity) to exert an impact on children’s core ASD symptoms. In other words, for autism-spectrum children with parents who reported more problems in regulating their own emotions, only those children with lower levels of resting cardiac vagal activity displayed more core symptoms such as social and communication deficits. This study provided one of the first physiological indications in support of autism literature, in which children with blunted resting cardiac vagal activity seemed to be a risk factor under the condition of parental emotion dysregulation.

First, the correlational analyses demonstrated no significant correlation between parental emotion dysregulation and children’s core symptoms. This exploration is a new endeavor in the ASD literature, with previous studies have mostly focused on parenting stress rather than parental characteristics in terms of their impact on their child (e.g., Keenan et al., 2017). However, it is still surprising given that emerging studies in typically developing children suggested the impact of parents’ own difficulties with ER on children’s social and emotional functioning (Morris et al., 2007). The absence of this finding in families of children with ASD compelled us to explore further potential moderating factors. As suggested by the risk and resilience framework (Masten, 2001), not all children with parents who reported more emotional difficulties would develop more core symptoms, there might exist protective and vulnerability factors. One such factor might be children’s physiological ER, indexed by cardiac vagal activity. As indicated by the polyvagal theory, the greater the resting cardiac vagal activity and the greater suppression of cardiac vagal activity in response to emotionally challenging events might reflect better ER. Our results demonstrated that the resting cardiac vagal activity, rather than cardiac vagal reactivity in response to a moderately stressful interaction task, moderated the relationship between parental emotion dysregulation and children’s core ASD symptoms.

Specifically, our results supported nascent ASD literature in that the resting cardiac vagal activity might be a reliable global measure of functioning in ASD children (Patriquin et al., 2013a,b). The majority of previous studies on cardiac vagal activity in ASD compared ASD with typically developing children on the resting cardiac vagal activity or the task-related changes of cardiac vagal activity (see Benevides and Lane, 2015 for a review). However, the results were generally mixed with some studies showing children with ASD demonstrating lower resting and task-specific cardiac vagal activity (e.g., Guy et al., 2014; Neuhaus et al., 2016), whereas others found no significant group difference (e.g., Condy et al., 2017). These mixed findings compelled us to consider that rather than conceptualizing cardiac vagal activity as a physiological deficit in children with ASD, the individual-level differences in cardiac vagal activity might serve as a protective/risk factor for these groups. Indeed, emerging studies suggested the connection between resting or task-specific cardiac vagal activity and a range of social and emotional functioning in ASD children (e.g., Edmiston et al., 2016; Condy et al., 2017). Such physiological characteristics might reflect children’s trait and conditional ER especially in aspects that cannot be captured through behavioral observation and subjective reports about children with ASD.

Interestingly, we found that only lower resting cardiac vagal activity, reflecting children’s poorer trait ER, served as a risk factor on the links between parental emotion dysregulation and children’s core ASD symptoms. For those children with ASD that demonstrated higher resting cardiac vagal activity, reflecting better trait ER, their core symptoms were not significantly influenced by their parents’ emotional functioning. Additionally, task-related changes of cardiac vagal activity, reflecting children’s conditional ER, did not significantly interact with parental emotion dysregulation on children’s core symptoms. These findings suggested that although cardiac vagal activity was recently conceptualized as a broad index of physiological functioning in children with ASD, it was important to consider resting and task-specific cardiac vagal activity as they reflected different aspects of child ER as well as had distinct functions for these children especially considering their family influence on their core symptoms.

It seemed that parental emotion dysregulation in their daily interaction with children with ASD, a negative aspect of parenting found in families with typically developing children (see Morris et al., 2007 for a review), was only harmful for those children with lower resting cardiac vagal activity (poorer trait ER). This study was among the first in ASD literature to identify a risk physiological factor for children in understanding family influences on their core symptoms. Future research on intervention of both parents’ and children’s ERs might improve the core symptoms of children with ASD. Moreover, potential intervention strategies could be provided to children with low HF-HRV in vagal nerve activation, such as HRV-biofeedback, with the purpose to assist children with ASD to be able to remain more resilient to the effects of their parents’ emotional regulation difficulties on their ASD symptoms. Despite the initiative contributions, our study was not without limitations. First, our primary limitation was that given the cross-sectional nature of the current design, we could not determine the direction of the effects. Future longitudinal designs with the measurement of cardiac vagal activity are strongly encouraged. Second, our study mainly focused on the resting and reactivity phases of child cardiac vagal activity. However, researchers have begun to emphasize the importance of cardiac vagal control recovery on individuals’ capacity for efficient self-regulation and adaptive functioning (e.g., Stanley et al., 2013; Laborde et al., 2017, 2018). Thus, to comprehensively understand the roles of different phases of cardiac vagal activity on individuals’ self-regulation, further studies are strongly encouraged to examine cardiac vagal control recovery together with measures of resting and reactivity. Additionally, taking different lengths of time to assess HRV (i.e., 2-min for resting session and 4-min during the task) might produce additional variances. Although 2-min resting recordings are acceptable when taking the sample characteristics into consideration (Malik et al., 1996), 5-min recordings for resting session are highly recommended (e.g., Malik et al., 1996; Laborde et al., 2017). Besides, measurements of cardiac vagal activity at rest and during the task are also recommended to be equivalent (Malik et al., 1996). To minimize the potential influences of different time lengths between measurements, in the current study, ECG data were parsed into 30-s epochs and were averaged to produce mean resting HRV and HRV reactivity, following suggestions of the Society for Psychophysiological Research Committee on Heart Rate Variability (Berntson et al., 1997). Further studies with longer resting session (i.e., 5 min) and equal lengths of measurements are encouraged to obtain more reliable ECG signals and accurate measurement of HRV during short-term recordings. Finally, although this study tried to incorporate multiple measures on study variables, future studies should include more informants (e.g., teachers, peers) and design situations in which the longer interaction between parents and children could be observed to examine the emotion dysregulations. Future studies with more standardized clinical assessment and diagnosis on ASD core symptoms would contribute to a better understanding of this association.

To conclude, although the current study did not find the direct association between parental emotion dysregulation and children’s core ASD symptoms, it supported the risk and resilience framework by providing one of the first physiological indications that the children’s blunted resting cardiac vagal activity worked as a risk factor on the relationship between parental emotion dysregulation and children’s core ASD symptoms. Together, this study suggested that the resting cardiac vagal activity might be a reliable global measure of physiological ER functioning in ASD children. It also added to the growing body of literature on the relationships among family influences, ER and social and behavioral functioning in children with ASD, suggesting the biofeedback training on ASD children’s physiological ER functioning might be a potential effective treatment especially for those who might be challenged by a negative familial emotional environment.

## Ethics Statement

This study was carried out in accordance with the recommendations of Institutional Review Board (IRB) of Faculty of Psychology, Beijing Normal University, China with written informed consent from all subjects. All subjects gave written informed consent in accordance with the Declaration of Helsinki. The protocol was approved by the Institutional Review Board (IRB) of Faculty of Psychology, Beijing Normal University, China.

## Author Contributions

XH, ZH, HW, and YH wrote the manuscript. ZH, QW, and LY designed the study and edited the manuscript. HW, YH, and SF collected and analyzed the data.

## Conflict of Interest Statement

The authors declare that the research was conducted in the absence of any commercial or financial relationships that could be construed as a potential conflict of interest.
